# Using diffraction losses of X-rays in a single crystal for determination of its lattice parameters as well as for monochromator calibration

**DOI:** 10.1107/S1600577521013667

**Published:** 2022-02-08

**Authors:** Nataliya Klimova, Irina Snigireva, Anatoly Snigirev, Oleksandr Yefanov

**Affiliations:** a International Science and Research Center ‘Coherent X-ray Optics for Megascience Facilities’, Immanuel Kant Baltic Federal University, Kaliningrad 236022, Russian Federation; b European Synchrotron Radiation Facility (ESRF), BP 220, 38043 Grenoble, France; c Center for Free-Electron Laser Science CFEL, Deutsches Elektronen-Synchrotron DESY, Notkestrasse 85, 22607 Hamburg, Germany

**Keywords:** X-ray glitches, diffraction losses, unit-cell parameter, single-crystal X-ray optics, monochromator calibration

## Abstract

A way has been developed to measure the unit-cell parameters of a single crystal just from an energy scan with X-rays, even when the exact energy of the X-rays is not well defined due to an error in the pitch angle of the monochromator. The precision of this measurement reaches *da*/*a* ∼ 1 × 10^−5^. The method is based on the analysis of diffraction losses of the beam, transmitted through a single crystal (the so-called ‘glitch effect’). This method can be easily applied to any transmissive X-ray optical element made of single crystals (for example, X-ray lenses). The only requirements are the possibility to change the energy of the generated X-ray beam and some intensity monitor to measure the transmitted intensity. The method is agnostic to the error in the monochromator tuning and it can even be used for determination of the absolute pitch (or 2θ) angle of the monochromator. Applying the same method to a crystal with well known lattice parameters allows determination of the exact cell parameters of the monochromator at any energy.

## Abstract 2

Diffraction losses (glitches) at certain energies of the X-ray beam, transmitted through a single crystal, can be used for lattice parameters determination as well as for calibrating the monochromator (absolute pitch angle and the unit-cell parameter).

## 1. Introduction

Effective optics are crucial to fully reveal the potential of fourth-generation synchrotron sources. Following their introduction in 1996 (Snigirev *et al.*, 1996 ▸), compound refractive lenses (CRLs) are commonly used for focusing X-rays at synchrotrons, due to easy alignment and good focusing efficiency. CRLs have since undergone development and adaptation for current and even new generations of synchrotron sources [MAX-IV (Tavares *et al.*, 2014 ▸), ESRF-EBS (ESRF, 2015–2022 ▸), PETRA IV (PETRA IV, 2019 ▸), SPring-8-II (Tanaka, 2016 ▸)], and X-ray free-electron lasers (XFELs) (Pellegrini, 2012 ▸) such as LCLS (Emma *et al.*, 2010 ▸) and the European XFEL (Altarelli, 2011 ▸).

CRLs are usually made of polycrystalline materials (for example, beryllium or aluminium) or single crystals (usually silicon, germanium or diamond). The optics made of single crystals usually have better properties: free of X-ray diffuse scattering from grain boundaries, voids, inclusions and other scattering centres, which reduces the amount of radiation in the focal spot. Hence, the best materials for the production of X-ray optics are single-crystal materials such as silicon, germanium and, even better, diamond.

However, we should note one drawback of X-ray optics made of single crystals – intensity modulation at certain energies in the transmission spectrum. This issue is termed ‘diffraction loss’ or the ‘glitch effect’ (Polikarpov *et al.*, 2018 ▸; Zhang *et al.*, 2019 ▸). The effect manifests itself as follows: at some energy of the X-rays, the transmitted (or diffracted in the case of monochromators) beam intensity drops. This happens when the diffraction condition is satisfied for some set of atomic planes in the crystal. Moreover, considering a potentially infinite number of sets of planes in the crystal, the probability of observing such ‘parasitic’ diffraction is rather high, especially for hard X-rays. This effect is particularly important for spectroscopic measurements, when a wide range of X-ray energies is scanned. Recently we have proposed a way to overcome this issue in some cases (Klimova, Snigireva *et al.*, 2021 ▸).

Nevertheless, we have found a way to use this drawback of single-crystal optics. In the present paper, we analyse the spectra measured previously (Polikarpov *et al.*, 2018 ▸; Zhang *et al.*, 2019 ▸) and we show that by careful analysis of glitches the unit-cell (UC) parameters of crystalline optics, used in transmission geometry, can be accurately determined. Usually, the UC parameters are determined from some diffraction experiments with X-rays or electrons – for a rather complete and recent review of the methods used for UC parameter determination see Lider (2020 ▸). Some of the methods can reach a precision of 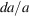 = 1 × 10^−9^ (Stoupin & Shvyd’ko, 2011 ▸; Toellner *et al.*, 2011 ▸). However, to get such high precision the absolute X-ray energy has to be known very accurately [for example, using a ^57^Fe Mossbauer radiation source (Lider, 2020 ▸)]. In addition, most of the methods require precise goniometers or rather complicated experimental geometry. Hence, some additional hardware modification of the beamline is usually needed.

We propose a way to determine the UC parameters of the optical element just from an energy scan which requires only an intensity monitor such as an ion chamber or a diode and, of course, the possibility to change the energy. The proposed method has a rather high precision ( 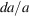 down to 1 × 10^−5^) and is indifferent to the errors in X-ray energy induced by the wrong monochromator pitch angle. Moreover, as we will demonstrate in this work, the proposed method can be used in a reciprocal way – to determine the absolute pitch angle of the monochromator. This is important for any application that requires exact knowledge of the absolute X-ray energy, especially for an experiment requiring changing energy (like spectroscopy).

Another issue that can lead to a wrong X-ray energy after the monochromator is its UC parameter. The X-ray monochromators are usually manufactured from high-quality silicon, germanium or diamond. The lattice parameter of these materials is well known to rather high precision. However, due to the heating induced by the beam, this parameter can change slightly (Petrov *et al.*, 2019 ▸). Usually, at least the first crystal of the X-ray monochromator is cooled with water or even liquid nitro­gen, so its temperature is supposed to stay stable. But for different X-ray energies the heat load changes as well as the penetration depth inside the monochromator crystal. Therefore, the effective UC parameter of the monochromator can change, leading to a wrong recalculation of the energy of the X-rays.

The method described in this paper can be used not only for the determination of the UC parameters of the studied single-crystal sample, but also in a reciprocal way. If the cell parameter of the sample is well known and the heating of the sample during the measurements is excluded (using an attenuated and unfocused beam), then the actual cell parameter of the monochromator can be determined at any energy with high precision.

All data processing described in this paper can be carried out automatically (Klimova *et al.*, 2020 ▸; Klimova, Yefanov *et al.*, 2021 ▸), and thus the method can be routinely used at most of the beamlines of modern synchrotrons. All the programs used for data processing are deposited as open-source projects at GitHub (https://github.com/XrayViz/Glitches).

## 2. Experimental observation of X-ray glitches in transmission geometry

**Figure 1:**
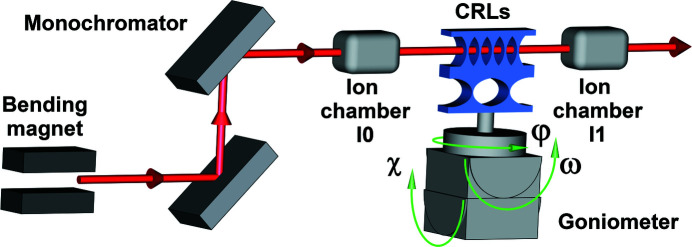
Experimental setup. The beam from the bending magnet is incident on the double-crystal monochromator. The intensity of the beam is measured with ion chambers before (I0) and after (I1) the sample. The sample (here a 1D CRL) can be rotated around all three axes.

We performed several X-ray spectroscopy measurements at the BM31 Swiss–Norwegian Beamlines (SNBL) of the European Synchrotron Radiation Facility (ESRF), France (Polikarpov *et al.*, 2018 ▸; Zhang *et al.*, 2019 ▸). At this beamline X-rays with a vertical size of 0.1 mm are generated by a bending magnet. Then the radiation passes through the double-crystal Si (111) monochromator and illuminates the sample. Two ionization chambers are installed before (I0) and after (I1) the sample to measure the incident and the transmitted intensities, respectively (Fig. 1 ▸).

The step of the energy scan was limited by the resolution of the monochromator of 1 eV. The sample was mounted on a three-cradle goniometer to measure the spectrum for different orientations of the sample with respect to the incident beam. More experimental details can be found in the work of Polikarpov *et al.* (2018 ▸) and Zhang *et al.* (2019 ▸).

**Figure 2:**
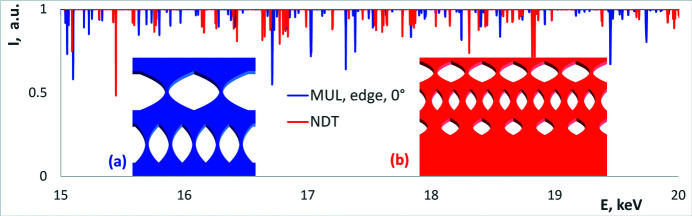
Normalized spectra of glitches for the two CRLs: blue (*a*) MUL and red (*b*) NDT. More details regarding the lenses can be found in the work of Polikarpov *et al.* (2018 ▸), Zhang *et al.*(2019 ▸).

To eliminate the glitches caused by the monochromator as well as to compensate the refills of the synchrotron ring with electrons, the transmitted intensity was normalized by the incident intensity (I1/I0) and then by its smoothed version (to compensate for the change in absorption by the sample with X-ray energy as well as different efficiency of the beamline and the ion chambers). The processing of the experimental spectra is automated and described in detail by Klimova *et al.* (2020 ▸), Klimova, Yefanov *et al.* (2021 ▸) and the programs are deposited at GitHub (https://github.com/XrayViz/Glitches). Examples of two normalized spectra of glitches for two CRLs are presented in Fig. 2 ▸.

During the beam time, the spectra of glitches of several different diamond samples were measured. The two 1D CRLs described in this paper are shown in the insets of Fig. 2 ▸. The first one consisted of two sets of CRLs machined by Micro Usinage Laser (MUL) (http://micro-usinage-laser.com/; Polikarpov *et al.*, 2015 ▸), Grattentour, France [Fig. 2 ▸(*a*)], and the second one consisted of three sets of CRLs manufactured by New Diamond Technology (NDT) (http://ndtcompany.com), Saint Petersburg, Russia [Fig. 2 ▸(*b*)]. More details regarding the measured samples can be found in the work of Polikarpov *et al.* (2018 ▸), Zhang *et al.* (2019 ▸), Klimova *et al.* (2020 ▸), Klimova, Yefanov *et al.* (2021 ▸).

## 3. Theory of glitch formation in transmission geometry

**Figure 3:**
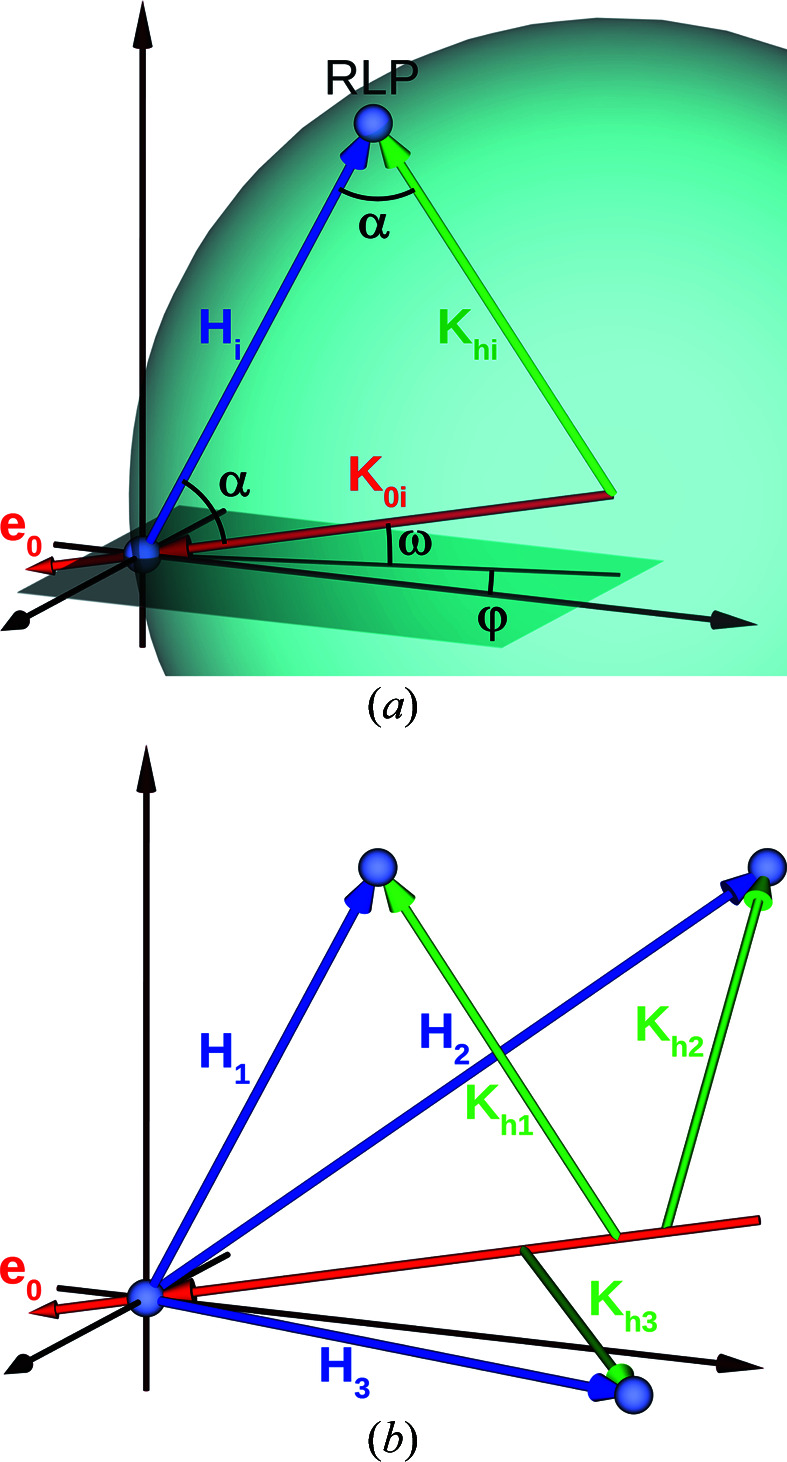
Diffraction in reciprocal space: (*a*) at a single RLP with the reciprocal vector **H**_*i*_. The incident beam *K*_0*i*_ along the unit vector **e**_0_ and the length of the diffracted beam is |**K**_*hi*_| = |**K**_0*i*_| = *K*_*i*_, where *K*_*i*_ is the radius of the Ewald sphere (blue) intersecting the excited RLP; (*b*) three different RLPs excited at different energies while the incident beam direction is kept constant (along the unit vector **e**_0_).

During the energy scan the direction of the beam, incident on the sample, is kept constant, while its energy (wavelength) changes. This means that in the reciprocal space the length of the incident wavevector *K*_0*i*_ is changing, while its direction (unit vector **e**_0_) is constant [Fig. 3 ▸(*a*)]. An animation demonstrating the process of glitch formation simulated using the program *XVis* (Yefanov *et al.*, 2008 ▸) can be found at GitHub (https://github.com/XrayViz/Glitches).

A glitch in the spectrum at energy *E*_*i*_ (or wavelength λ_*i*_) is formed when the Ewald sphere with radius

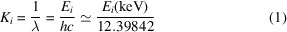

and centred at the beginning of the current vector **K**_0*i*_ intersects some reciprocal-lattice point (RLP) characterized by the reciprocal-lettice vector **H**_*i*_. From the isosceles triangle formed by the vectors **H**_*i*_, **K**_0*i*_, **K**_*hi*_ [Fig. 3 ▸(*a*)] one can determine the cos(α):

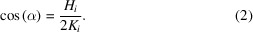

At the same time the scalar multiplication is

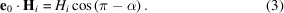

Hence, one can write cos(α) as follows:

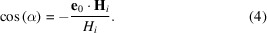

Combining equations (2) and (4) one can determine the *K*_*i*_:

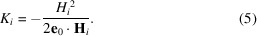

Finally, considering equation (1), the energy of the glitch is

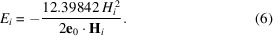

From equation (6) it is obvious that the energy of each glitch depends on the orientation of the incident beam with respect to the crystalline lattice (vector **e**_0_) and the reciprocal-space vector **H**_*i*_, which depends on the UC parameters (*a*, *b*, *c*, α, β, γ in the general case). We consider the cubic cell as the one most commonly used for X-ray optics, but the method described here can be applied to a general case with some modifications. For a cubic cell only one parameter is important: the unit-cell size *a*. Let us redefine the reciprocal vector **H**_*i*_ to be dimensionless:

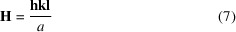

where **hkl** is a dimensionless vector made of the corresponding *h*, *k*, *l* Miller indices.

Substituting equation (7) into equation (6) one gets

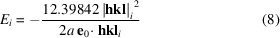

where 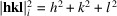 .

As can be seen from equation (8), the energy of the glitch depends not only on the crystal lattice orientation but also on the UC parameter. In addition, as will be shown later, the spectrum of the glitches is very sensitive to these two parameters (orientation and cell size).

From equation (8) the energy of each glitch can be found, if the orientation of the incident beam with respect to the crystalline lattice of the sample is known. The reciprocal task can also be solved: knowing the glitch spectrum of a sample, its orientation can be determined. Hence, at this stage, we have to solve such a reciprocal problem. However, first, we need to determine which measured glitch corresponds to which RLP of the crystal. This problem is quite similar to one of the tasks in crystallography, termed the ‘indexing problem’: when a Miller index has to be found for each Bragg peak at a measured diffraction pattern. Therefore, to find an approximate solution we define some orientation of the crystal to the incident beam [only two angles ω and φ, see Fig. 3 ▸(*a*), are important because the rotation around the beam does not change diffraction conditions] and the UC parameter and calculate the energies of all possible glitches (for allowed reflections) using equation (8). Then the difference between the calculated energies 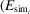 ) and all measured ones 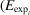 ) is

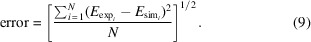

By minimizing the error described by equation (9) one can determine an approximate orientation of the sample lattice with respect to the incident beam as well as its UC parameter.

As already mentioned, there are only three parameters to identify: angles ω and φ (between the beam and the crystalline lattice) and the UC parameter *a*. Having measured only three glitches (for the cubic cell) one can determine all three parameters. The precise solution through fitting could be rather time-consuming. Therefore, we propose to perform it in two steps: first, we determine the orientation and the UC parameter approximately, to be able to ‘index’ the glitches (attribute Miller indices to each glitch), and then we refine the parameters using an analytical approach.

### 3.1. Refining the orientation and UC parameters using an analytical approach

Let us consider three glitches *E*_1,_ *E*_2,_ *E*_3_ with the corresponding Miller indices *h*_1_ *k*_1_ *l*_1_, *h*_2_ *k*_2_ *l*_2_, *h*_3_ *k*_3_ *l*_3_ [see Fig. 3 ▸(*b*)] – after the ‘indexing’ the correspondence of each glitch energy to the Miller indices is known. For these three reflections equation (8) can be written as a system of equations (Klimova *et al.*, 2020 ▸):

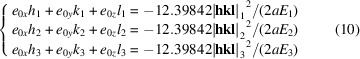

or in a matrix form:

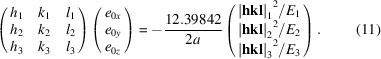

The matrix equation (11) is linear with respect to the incidence unit vector **e**_0_ with an unknown scaling coefficient 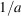 . However, considering that the absolute value of any unit vector is unity, the UC parameter *a* can be easily determined. In the case of many measured glitches, equation (11) becomes overdetermined and can be solved using, for example, numerical methods (https://github.com/XrayViz/Glitches).

## 4. Experimental data processing

As described earlier, the measured spectrum is normalized (Fig. 2 ▸) and then the actual energy of each glitch has to be determined. We extract the energy of glitches using the automatic approach described in detail by Klimova, Yefanov *et al.* (2021 ▸) – the corresponding program is deposited at GitHub (https://github.com/XrayViz/Glitches).

The energies of the glitches are processed in two steps: first by fitting the approximate orientation and UC parameter to index the measured glitches and then refining the parameters by solving the system of equations (11). The sources of the programs for this part of data processing are also deposited at GitHub (https://github.com/XrayViz/Glitches). We should note here that in our previous work (Klimova, Yefanov *et al.*, 2021 ▸) we have used a different approach for the indexing: we have probed all energies for each orientation to find the RLPs that are close to the Ewald sphere for each energy. Our current approach described in Section 3 is approximately 1000 times faster.

**Figure 4:**
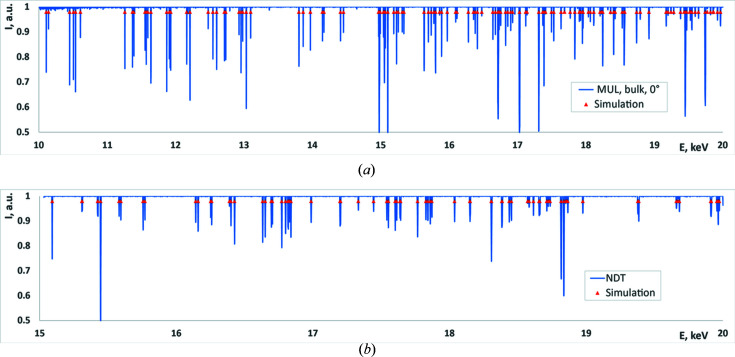
Spectra of measured glitches (blue curves) overlapped with the calculated spectra (red triangles) for two 1D lenses: (*a*) MUL and (*b*) NDT.

Some results of the processed spectra of glitches are presented in Fig. 4 ▸. More results for different samples measured in different configurations can be found in the work of Klimova, Yefanov *et al.* (2021 ▸).

## 5. UC determination regardless of the monochromator error

**Figure 5:**
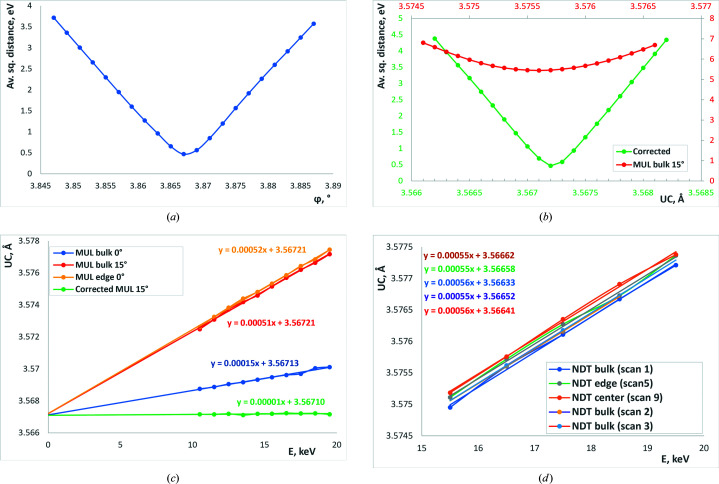
Sensitivity of glitches to (*a*) angle φ and (*b*) UC parameter (red, before the energy correction; green, after). The apparent dependence of UC parameters on energy for (*c*) MUL lens (blue line is the scan before the monochromator re-tune, red and yellow lines after the re-tune, and the green line is the same data as the red line but after the energy correction) and for (*d*) NDT lens (different scans).

The glitch spectra are very sensitive to the orientation of the lens and to the UC parameter of the single crystal [see Figs. 5 ▸(*a*), 5 ▸(*b*)]. Even a small change in the UC parameter or orientation is reflected in the error [equation (9)] in the glitch’s determination. This leads to the fact that usage of glitches allows the accurate determination of the parameters of the crystal. One can estimate the precision from the plots shown in Figs. 5 ▸(*a*), 5 ▸(*b*): the easily detectable change of the error by 10% corresponds to the change of the angle by approximately 0.0005° or the change of the UC parameter of 0.0001 Å.

We have used the sensitivity of the glitches to the orientation and the UC parameter to determine the orientation of each measured sample – see Klimova, Yefanov *et al.* (2021 ▸) for details. However, during the data processing, we have found the following effect: it looked as though the UC parameters of the lenses were growing with the energy of the X-ray beam [Figs. 5 ▸(*c*), 5 ▸(*d*)]. The change of the UC parameter can be approximated by the line [Fig. 5 ▸(*c*), 5 ▸(*d*)]

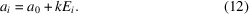

After careful analysis, we have figured that the determination of the different UC parameters was due to the wrong calibration of the monochromator at the beamline. In Fig. 5 ▸(*c*) the samples measured on different days (before and after re-tuning of the beamline) have different cell dependence on energy (red and yellow line versus blue line). However, at 0 eV all lines intersect giving the true UC parameter (*a*_0_) that is, in this case, 3.56718 ± 0.00005 Å. For the second measured lens (NDT) we determined the cell parameter to be 3.5665 ± 0.0002 Å – the precision is worse due to the small energy scans for this sample (2–5 keV for the NDT compared with 10 keV for the MUL). Both cell parameters are very close to the published value which is 3.567 Å. Hence, even with the wrong energy calibration of the beamline, we managed to recover the true UC parameters of the crystal.

## 6. Tuning monochromator pitch angle using glitches

The theory of glitches, described in this paper, works only if the absolute energy of X-rays, emerging from the monochromator, is known with high precision. Unfortunately, quite often it is not the case. The most common issues leading to wrong monochromator energy calibration are: offset in the pitch (or 2θ) angle or change in the cell parameter (*a*_mono_) of the monochromator crystal, for example, due to heating. As will be demonstrated later, the second effect leads to the scaling of the determined cell parameter. The first issue (wrong determination of the absolute pitch angle) leads to a nonlinear discrepancy in the determination of the energy through the measured spectrum.

Considering these effects, the energy of the beam, after the monochromator, is calculated using Bragg’s law:

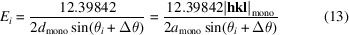

where *d*_mono_ is the interplanar distance and *a*_mono_ the UC parameter of the monochromator, with Miller indices *h*_mono_, *l*_mono_, *k*_mono_, so 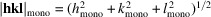 , and Δθ is the error in the absolute value of the pitch angle θ_*i*_ of the monochromator.

Combining equations (9), (12) and (13) one can approximately derive the pitch offset:

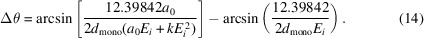

The angular error Δθ changes slowly with the energy *E*_*i*_, so *E*_*i*_ can be chosen as the mean energy of the scan.

It is even better to calculate the angular offset Δθ while solving the system of equations (11). Considering equation (13), every equation in (10) can be written as

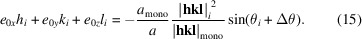

The nonlinear system of equations (15) can be solved numerically, or rewritten as a linear system:

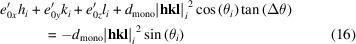

with four unknowns: 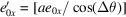 , 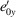 = 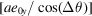 , 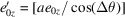 , tan(Δθ); thus to solve equation (16) at least four measured glitches are necessary.

Unfortunately, the angular error Δθ can lead to the wrong indexing of the measured glitches – wrong Miller indices can be assigned to some of the glitches. Therefore, it is better to refine the angular offset Δθ at the indexing stage – this would make the indexing process longer, but the whole procedure will be more reliable. The corresponding program can also be found at GitHub (https://github.com/XrayViz/Glitches). Alternatively, the approximate angular correction can be determined using equation (14), then the experimental spectrum is recalculated and the indexing–refinement procedure repeated.

**Figure 6:**
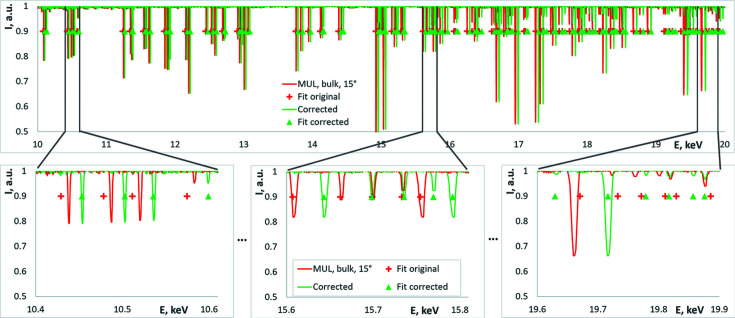
The glitch spectra before (red) and after (green) the energy recalibration (top plot). The green triangles and red crosses represent the simulated glitches for the two spectra. To see the details, small parts of the spectrum are presented over the whole measured range (10–20 keV) on the bottom row.

The procedure of the monochromator pitch angle correction led to the following corrections in our measurements: Δθ was −0.0164° and −0.0045° for the two cases shown in Fig. 5 ▸(*c*) which corresponded to an error in energy determination of 44 eV and 12 eV, respectively, at 10 keV range. Fig. 6 ▸ demonstrates the energy spectra before and after the correction overlapped with the simulated spectra of glitches in both cases.

As can be seen from Fig. 6 ▸, the best fit for the original data is rather good only in the middle of the scan, while at the edges of the energy range the fit is quite poor (average square error for the whole spectrum was 5.47 eV). At the same time, after the correction, the whole range is fitted very well (with average square error of 0.465 eV).

We should note that the difference between the spectra before and after correction in Fig. 6 ▸ is not only the scaling of the spectrum, otherwise it would be predicted by the change of the UC parameter [see equation (9)]. However, the change of the offset of the monochromator 2θ angle (pitch) allows us to fully correct the spectrum. Therefore, we can conclude that the method presented in this work can be used for unambiguous calibration of the pitch (2θ) angle of the monochromator.

## 7. Refining the monochromator UC parameter using glitches

There is one more very useful consequence from equation (15). The UC parameter of the studied sample *a* and the UC parameter of the monochromator *a*_mono_ are tightly connected – only the ratio 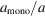 is important [see equation (15)]. Therefore, if the UC parameter of the studied sample is well known, it is possible to determine the cell parameter of the monochromator with high accuracy.

As was mentioned in Section 1, the determination of the UC parameter of the monochromator at current X-ray energy is quite an important task. We should note here that usually a monochromator consists of several crystals. Also, due to the heat dissipation inside the first crystal there could be a cell parameter gradient (Petrov *et al.*, 2019 ▸) which also influences the energy of the beam. Therefore, the UC parameter that we determine is the ‘effective’ parameter – this is the parameter that is used in the Bragg equation to determine the current energy. Therefore, this is actually the same parameter that we need to know for correct energy recalculation.

A way to perform such calibration using glitches is as follows. A calibration sample with well known cell parameter (for example, a thin slab of high-quality silicon crystal) is installed in the beam in transmission geometry. The monochromator is tuned to the desired energy and the sample is illuminated by the unfocused and attenuated beam to prevent heating of the sample. Then a small energy scan is performed and the intensity before and after the sample is measured. If there is no possibility of measuring both intensities simultaneously, one can measure the spectrum twice: with and without the sample. The energy range of the measured spectrum at hard X-rays (>10 keV) can be rather small – 200 eV should be sufficient. For lower energies a larger scan could be needed – the programs to simulate the glitches for a crystal with a diamond cubic lattice (silicon, germanium, diamond) can be found at GitHub (https://github.com/XrayViz/Glitches). From the measured spectrum, using the approach described in this paper, one can calculate the pitch offset Δθ and the monochromator cell parameter *a*_mono_.

There is one more way to perform such a calibration. If the beamline is equipped with a precise goniometer, but the energy scans are not possible, one can measure intensity drops of the transmitted beam while rotating the calibration crystal. Knowing the relative angles between the glitches measured in such a way, one can also calibrate both pitch angle and cell parameter of the monochromator for any energy. A detailed description of this idea, together with an experimental demonstration, will be published separately.

## 8. Conclusion

In the present paper, we have demonstrated a method to determine UC parameters of any single crystal with a cubic cell using its spectra of glitches. We have shown that the method is quite sensitive – it allows one to determine the changes in the UC parameter of just 1 × 10^−4^ Å. This method can be really useful to find the exact UC parameters of X-ray optics used at modern beamlines at synchrotrons or free-electron lasers. The main advantage of the method is the fact that the configuration of the beamline does not have to be changed – the only requirements are the possibility to scan the wavelength of the incident beam (spectroscopy mode) and some intensity monitor. In our experiment, we used two intensity monitors: one before the sample and one after. However, in principle, one intensity monitor is enough, just used twice (with and without the sample). The method allows one to determine the UC parameters of the transmissive crystalline optics in the same condition as is used at the beamline, for example, considering the heating caused by the X-ray beam.

One more advantage of the proposed method is its robustness to the error in the monochromator calibration. A badly defined pitch (or 2θ) angle of the monochromator does not influence the UC parameter determination. In fact, the whole method can be used for calibrating the energy at any beamline, where an energy scan is possible, by determining the absolute pitch (2θ) angle of the monochromator. Moreover, this can be performed even for past spectroscopic experiments by analysing the measured data, if the range of the energy change is sufficient to determine at least four glitches. The extension of this method, using only the glitch spectrum of the monochromator, will be presented in our next paper.

The final outcome of the proposed method, of using glitches, is the possibility of determining the ‘effective’ UC parameter of the monochromator for the current energy of the generated X-rays. This is important, because the UC parameter of the first monochromator crystal can change due to the heating induced by the beam. To determine the true UC parameter of the monochromator, a glitch spectrum of a calibration sample with well known UC parameter can be measured and analysed. Then, knowing the UC parameter of the sample, one can easily calculate the true UC parameter of the monochromator. Therefore, the main sources of errors in the monochromator calibration (wrong pitch angle and the UC parameter) can be eliminated using only a small glitch spectrum from a well known sample.
